# Neuroprotection against Amyloid-*β*-Induced DNA Double-Strand Breaks Is Mediated by Multiple Retinoic Acid-Dependent Pathways

**DOI:** 10.1155/2020/9369815

**Published:** 2020-03-20

**Authors:** Julien Colas, Natacha Chessel, Allaeddine Ouared, Emmanuelle Gruz-Gibelli, Pascale Marin, François R. Herrmann, Armand Savioz

**Affiliations:** ^1^Department of Psychiatry, Division of Geriatric Psychiatry, University Hospital of Geneva, Geneva, Switzerland; ^2^Department of Rehabilitation and Geriatrics, Division of Geriatrics, University Hospital of Geneva and University of Geneva, Geneva, Switzerland; ^3^Geneva University Neurocenter, University of Geneva, Geneva, Switzerland

## Abstract

In this study, we have investigated the role of all-*trans*-retinoic acid (RA) as a neuroprotective agent against A*β*_1-42_-induced DNA double-strand breaks (DSBs) in neuronal SH-SY5Y and astrocytic DI TNC_1_ cell lines and in murine brain tissues, by single-cell gel electrophoresis. We showed that RA does not only repair A*β*_1-42_-induced DSBs, as already known, but also prevents their occurrence. This effect is independent of that of other antioxidants studied, such as vitamin C, and appears to be mediated, at least in part, by changes in expression, not of the RAR*α*, but of the PPAR*β*/*δ* and of antiamyloidogenic proteins, such as ADAM10, implying a decreased production of endogenous A*β*. Whereas A*β*_1-42_ needs transcription and translation for DSB production, RA protects against A*β*_1-42_-induced DSBs at the posttranslational level through both the RAR*α*/*β*/*γ* and PPAR*β*/*δ* receptors as demonstrated by using specific antagonists. Furthermore, it could be shown by a proximity ligation assay that the PPAR*β*/*δ*-RXR interactions, not the RAR*α*/*β*/*γ*-RXR interactions, increased in the cells when a 10 min RA treatment was followed by a 20 min A*β*_1-42_ treatment. Thus, the PPAR*β*/*δ* receptor, known for its antiapoptotic function, might for these short-time treatments play a role in neuroprotection via PPAR*β*/*δ*-RXR heterodimerization and possibly expression of antiamyloidogenic genes. Overall, this study shows that RA can not only repair A*β*_1-42_-induced DSBs but also prevent them via the RAR*α*/*β*/*γ* and PPAR*β*/*δ* receptors. It suggests that the RA-dependent pathways belong to an anti-DSB Adaptative Gene Expression (DSB-AGE) system that can be targeted by prevention strategies to preserve memory in Alzheimer's disease and aging.

## 1. Introduction

Recently, there was an increased interest in the involvement of cell nuclear changes in Alzheimer's disease (AD) as most of its key players have been shown to be involved either in gene expression, e.g., ApoE4 [1], or in DNA alterations, e.g., the Tau protein [2]. It has been shown that the A*β*_1-42_ peptide—in this paper referred to as A*β* peptide—not only regulates gene transcription [3, 4] but also impairs DNA repair [5]. Furthermore, the A*β* peptide generates DNA double-strand breaks or DSBs [6, 7] through oxidative stress [8], through depletion of the DNA repair factor BRCA1 [9], and through activity reduction of the DNA-dependent protein kinase (DNA-PK) [10, 11], a key enzyme of the nonhomologous end joining pathway (NHEJ) involved in DSB repair in neurons [12]. In this context, it is worth knowing that DSBs within promoter regions have been shown to be needed for transcription of neuronal early-response genes [13] or to play a role in gene silencing [14]. DSBs are involved in physiological processes, such as in memorisation [6], in aging [15–17], and in AD [16, 18–23].

Several factors playing a role against DNA damages have been identified, such as glutamine against etoposide-induced damages [24] or histones against DNA oxidation [8]. Some of them are known in relation to AD. Thus, monomeric Tau can protect DNA against heat shock-induced damages [25, 26] and NAD against A*β*-induced damages [7]. Recently, we showed in astrocytic DI TNC_1_ and neuroblastoma SH-SY5Y cell lines, as well as in the murine neocortex, that the vitamin A derivative all-*trans*-retinoic acid (RA) and the RAR*α*/*β* agonist Am80 are involved in the repair of A*β*-induced DSBs [27], RA or Am80 being added after A*β* in these experiments. The DNA-dependent protein kinase (DNA-PK) of the NHEJ pathway and Ataxia Telangiectasia Mutated kinase (ATM) were shown to be implicated as repair factors [27]. This represents an additional effect of RA besides the already known ones as a neuroprotective agent for Alzheimer's disease [28–31].

RA does not only repair A*β*-induced DSBs but also decreases the A*β* peptide production from the Amyloid Precursor Protein or APP. Indeed, RA can increase, via the RAR*α* receptor, the expression of ADAM10 (a disintegrin and metalloproteinase domain-containing protein 10), the major *α*-secretase [32], more than it increases the *β*-site cleaving enzyme BACE1, a *β*-secretase [33]. Furthermore, RA achieves a similar result by inhibiting the *γ*-secretase (or Presenilin-1) activity through activation of the Extracellular Signal-Regulated Kinase (ERK) 1/2 [34]. Thus, by changing the expression of enzymes of the amyloid cascade, RA might also prevent the formation of A*β*-induced DSBs and be a factor of neuroprotection against A*β*-induced DSBs and not just of DSB repair. The aim of the present study was to demonstrate that indeed RA not only repairs A*β*-induced DSBs, as described in our previous publication [27], but also prevents their occurrence. For such a purpose, neuroprotective experiments have been carried out with RA added before or at the same time as A*β* and not after as in repair experiments.

Overall, we propose that RA can protect DNA against A*β*-induced DSBs potentially allowing prevention strategies. We also show that it can protect posttranslationally through both the RAR*α*/*β*/*γ* and PPAR*β*/*δ* receptor-dependent pathways [35, 36] and in part via PPAR*β*/*δ*-RXR heterodimerization.

## 2. Materials and Methods

### 2.1. Murine Brain Tissues and Dissections

One- to 4- and 16- to 17-month-old C57BL/6J male mice (Janvier, Le Genest-St-Isle, France) were sacrificed by CO_2_ inhalation, and their brains were isolated. Either all cortical layers or the superficial (I–III) and deep (V-VI) neocortical layers, separated under a binocular microscope at the level of layer IV, were used. In all cases, the most rostral and caudal cortical parts were excluded. These tissues, as well as the hippocampus, were dissected in PBS and minced. The treatments with 5 *μ*M RA (Sigma-Aldrich), 20 *μ*M monomeric A*β*_1-42_ peptides (Enzo Life Sciences), and RA+A*β* were immediately carried out a in neurobasal medium (Gibco Life Technologies) in the presence of Penicillin G (100 IU/mL)/Streptomycin (100 *μ*g/mL; Invitrogen) and 2 mM L-glutamine (Gibco Life Technologies) for 30 min at 37°C in a CO_2_ incubator containing 5% CO_2_ and 95% humidified air. After a centrifugation step (300 rpm, 3 min), the supernatant was discarded, the pellet was mixed mechanically in PBS in the presence of 2 mM EDTA, and the cells counted for the comet and the cell viability assays.

For each treatment condition, three mice were sacrificed in accordance with Federal Swiss Veterinary regulations and institutional approval.

### 2.2. Cell Culture

SH-SY5Y cells (European Collection of Animal Cell Culture, UK) were grown in a CO_2_ incubator at 37°C in RPMI-1640 (Gibco Life Technologies) with 10% FCS (Bioconcept, Switzerland), Penicillin/Streptomycin, and L-glutamine. Once at confluence, cells were released in DPBS (150 mM NaCl, 3 mM KCl, 1.5 mM KH_2_PO_4_, 7.9 mM Na_2_HPO_4_·2H_2_O, 0.1 mM EDTA, pH 7.4), centrifuged 5 min at 1000 rpm, and resuspended at the desired dilution in RPMI-1640 with 10% FCS.

DMEM (Sigma) supplemented with 10% FCS was used to grow the DI TNC_1_ astrocytic cells [37, 38]. Once at confluence, they were released in trypsin solution (0.25%), centrifuged 5 min at 1100 rpm, and resuspended at the desired dilution in DMEM with 10% FCS.

### 2.3. Cell Treatments

For the cell treatments, the 10% FCS medium was replaced two days before the experiment by the 1% FCS medium. Cultured cells were treated or not, in a 37°C incubator containing 5% CO_2_ and 95% humidified air for 30 min with 1 *μ*M RA, for the Western blots, to 5 *μ*M RA, then after medium change, for further 30 min with 20 *μ*M A*β* peptides or not (ø) resulting in four combinations of 2 × 30 min treatments (ø–ø, ø–A*β*, RA–ø, and RA–A*β*). Treatments were also carried out simultaneously, and RA replaced by 5 *μ*M 9-cis RA (Sigma-Aldrich), 1 *μ*M glutathione (Sigma-Aldrich), 1 *μ*M *α*-tocopherol, 100 *μ*M carnosine (Sigma-Aldrich), or 100 *μ*M vitamin C (Sigma-Aldrich). Furthermore, 1 *μ*g/mL transcription inhibitor actinomycin D (Sigma-Aldrich), 35 *μ*M translation inhibitor cycloheximide (CHX; Sigma-Aldrich), 50 *μ*M RAR*α*/*β*/*γ* antagonist AGN 193109 (Abcam), and 10 *μ*M PPAR*β*/*δ* antagonist GSK 3787 (Santa Cruz Biotechnology) were added or not, in combination with RA and/or A*β* for 30 min.

### 2.4. Neutral Single-Cell Gel Electrophoresis (Comet Assay)

The Trevigen Comet Assay™ kit (AMS Biotechnology, UK) was used with SH-SY5Y or DI TNC_1_ cells, and the homogenized cortical tissues with the following modifications. Subsequently to the treatments, 1.0 to 1.5 × 10^5^ cells/mL were resuspended in ice-cold Ca^2+^- and Mg^2+^-free PBS. For the dissociated tissue, 20 mM EDTA was added to an equivalent number of cells in ice-cold Ca^2+^- and Mg^2+^-free PBS and processed as the cultured cells. 50 *μ*L cells were mixed with 500 *μ*L of 1% low-melting agarose (Seaplaque, FMC BioProduct, USA) kept at 42°C, and 50 *μ*L of the mixture was immediately added to a comet slide (AMS Biotechnology), put in the dark at 4°C for 10 min to promote agarose gelling and then at 4°C for 60 min in prechilled lysis solution (AMS Biotechnology). DSBs were separated by electrophoresis for 45 min at 26 V following an incubation step of the slides in Neutral Electrophoresis Buffer (100 mM Tris base, 0.3 M sodium acetate, pH 9.0) at 4°C for 30 min. The slides were subsequently immersed in 70% ethanol at room temperature for 30 min and air dried. DNA was stained with 100 *μ*L SYBR Green I dye (Gibco Life Technologies) diluted 1 : 1000 in water for 10 min and then rinsed with distilled water, all at room temperature. Generally, at least 30 comets per treatments were photographed with an Olympus digital camera attached to an epifluorescent Zeiss Axioplan microscope (Axio vision rel 4.6). The length of one comet was obtained by subtracting the diameter of the cell body (perpendicular to the orientation of the electrophoresis) from the total signal length (parallel to the orientation of the electrophoresis) consisting of the length of the cell body and of the comet tail. This value was then converted in *μ*M according to the magnification factor of the microscope.

### 2.5. Western Blot

For the Western blots, the dissected tissue was weighted, homogenized, and treated during different times in the neurobasal medium with Penicillin/Streptomycin and L-glutamine. After a centrifugation step at 1200 rpm for 5 min, the supernatant was discarded and 600 *μ*L of lysis buffer 1x (50 mM Tris, 2% SDS, 5 mM EDTA, and 2 mM EGTA, pH 6.8) per 20 mg tissue, 125 nM okadaic acid, and protease inhibitor cocktail diluted 1 : 100 (Sigma) were added. Each sample was finally sonicated. The protein concentrations were quantified by the BCA protein assay (Pierce, Thermo Scientific), and 20 *μ*g of proteins were diluted in Laemmli loading buffer (20 mM Tris-HCl, pH 6.8, 1% SDS, 7% glycerol, 2.0% *β*-mercaptoethanol, 0.05% bromophenol blue), denatured (100°C, 5 min), separated (Mini-Protean TGX precast gels, Bio-Rad, 60 V, 1 h, then 90 V, 1 h), and electroblotted overnight (54 V; Mini Trans-Blot electrophoretic transfer cell, Bio-Rad) to a nitrocellulose membrane (Protran BA85, Schleicher and Schuell) according to a standard Western blot method [39]. 6.5 *μ*L Seeblue Plus 2 prestained protein molecular weight markers (Invitrogen) were loaded. The membranes were incubated overnight at 4°C in PBS, 0.2% Tween 20, 0.5% BSA, and 5% milk with the following primary rabbit polyclonal antibodies: anti-ADAM10 (ab1997, Abcam); anti-APP, C-terminal (AICD, A8717, Sigma-Aldrich); anti-BACE1 (#5606, Cell Signalling Technology); anti-GluN2B (NR2B; ab65783, Abcam); anti-Peroxisome Proliferator-Activated Receptor *β*/*δ* (PPAR*β*/*δ*, H-74, sc-7197, Santa Cruz Biotechnology); anti-Presenilin-1 (H-70, sc-7860, Santa Cruz Biotechnology); anti-PSD95 (PA1-4667, Thermo Fisher Scientific); and anti-RAR*α* (PA1-810A, Thermo Fisher Scientific). The following primary mouse monoclonal antibodies were used: AT8 (MN1020, Thermo Fisher Scientific) and Tau1 (MAB3420, Millipore). All primary antibodies were diluted 1 : 1000 except when specified otherways. The horseradish peroxidase-labeled anti-rabbit or anti-mouse antibodies (DakoCytomation) were diluted 1 : 3000 in PBS with 0.2% Tween 20, 0.5% BSA, and 5% milk and used as secondary antibodies.

The Amersham ECL™ Western Blotting Detection Reagent (GE Healthcare, Buckinghamshire, UK) on Hyperfilm ECL (Amersham Biosciences, GE Healthcare) was used to detect specific signals. Mouse monoclonal anti-GAPDH (Millipore; 1 : 10000) was used to verify equal protein loading as well as DB71 protein staining of the blotting membranes (Aldrich, Milwaukee, USA). The ratios of the signals for a defined protein, measured by densitometry (U:Genius 3 with GeneTools from Syngene) and divided by the DB71 or GAPDH signals, were used for statistical analysis.

### 2.6. Immunocytology

After removal of the cell medium, SH-SY5Y cells were treated or not with 5 *μ*M RA for 10 min and then for 20 min with 20 *μ*M A*β* or not, rinsed 5 min in PBS, fixed 30 min in 4% paraformaldehyde, and finally washed again 5 min in PBS. After 10 min permeabilisation in PBS in the presence of 0.2% Triton X-100, the cells were washed twice as above and incubated 90-120 min at room temperature in PBS with the primary antibody against PPAR*β*/*δ* (H-74, sc-7197, Santa Cruz Biotechnology, 1 : 50) and the one against RXR*α*/*β*/*γ* (C-20, sc-831, Santa Cruz Biotechnology, 1 : 25), or the antibody against RAR*α*/*β*/*γ* (sc-366090, Santa Cruz Biotechnology, 1 : 100) and the one against RXR*α*/*β*/*γ*. The cells were then washed twice, incubated 1 h at room temperature with the secondary fluorescent anti-goat or anti-rabbit antibodies (Alexa, 1 : 1000), rinsed again 5 min in PBS, colored 5 min in 0.1% DAPI, and finally mounted in fluorosave. Omission of the primary antibodies resulted in no immunopositive signal over background. Analyses were carried out with an epifluorescent Zeiss Axioplan microscope.

### 2.7. Duolink™ In Situ Proximity Ligation Assay (PLA)

Cells were treated or not with 5 *μ*M RA for 10, 30, or 60 min and then for, respectively, 20, 30, or 60 min with 20 *μ*M A*β* or not. They were subsequently deposited on glass slides previously sterilized with alcohol 100%. They were fixed in PAF 4% during 20 min, washed in 1x Duolink *in situ* Wash Buffer A (Sigma), and incubated in a drop of Duolink blocking solution for 30 min. Then, the primary antibody anti-RAR*α* (PA1-810A, Thermo Fisher Scientific) or anti-PPAR*β*/*δ* (H-74, sc-7197, Santa Cruz Biotechnology) was pooled with anti-RXR*α*/*β*/*γ* (C-20, sc-831, Santa Cruz Biotechnology) after having been diluted in Antibody Diluent (Sigma). Both antibodies were incubated 1 h and 30 min at room temperature. Subsequently, the cells were washed with PBS 1x (1 mL/well). The two PLA probes, *in situ* PLA Probe anti-rabbit Plus and *in situ* PLA Probe anti-mouse Minus, diluted 1 : 5 in 80 *μ*L primary antibody solution and kept for 20 min at room temperature, were added. The samples were incubated in a preheated humidity chamber for 1 h at 37°C. They were washed with 1x Wash Buffer A for 2 × 5 min under gentle agitation and 80 *μ*L ligation solution diluted 1 : 40 (1 U/*μ*L) were added and incubated for 30 min at 37°C. After removal of the ligation solution, the cells were washed as before in the Wash Buffer A but for 2 × 1 min, and the polymerase (10 units/*μ*L) was diluted 1 : 80 in the amplification solution, added, and incubated in a preheated humidity chamber for 100 min at 37°C. Afterwards, the solution was removed and the cells washed with 1x Wash Buffer B (Sigma) for 2 × 2 min and subsequently with 0.01x Wash Buffer B for 1 min. Then, the slides were dried at room temperature in the dark, mounted with a minimal volume of FluorSave (Calbiochem) with DAPI (1/5000), and, after 5 min, analysed with an epifluorescent Zeiss Axioplan microscope.

### 2.8. Statistical Analysis

Values of mean comet tail length were compared for each experiment by a one-way analysis of variance (ANOVA). When overall statistically significant treatment differences were reached by ANOVA, comparisons of means among the subgroups were calculated with Bonferroni corrections. In parallel, the nonparametric Kruskal-Wallis test was used to compare the shape of comet tail distribution also with Bonferroni corrections to compare subgroups. For the Western blots, gene expression was compared using two-way repeated measures ANOVA, with age (young versus old) and treatment effect (without RA and 0.5 h, 1 h, 2 h, and 3 h with RA) along with their interaction. Treatment was the within-subject factor. The same analysis was performed separately for measures of the superficial and deep cortical layers, for each protein (e.g., PSD 95, NR2B) normalized with the control used (DB71 or GAPDH). The significance of values was determined using the Greenhouse–Geisser method. The level of significance is *p* < 0.05. Analyses were carried out with the Stata 14.1 software (Stat Corp., TX, USA, 2013). Alternatively, we used the program Prism 7 (GraphPad).

## 3. Results

### 3.1. Retinoic Acid Protects against A*β*-Induced DSBs in SH-SY5Y and DI TNC_1_ Cells as well as in the Neocortex of Young and Aged C57BL/6J Mice

**Figure 1:**
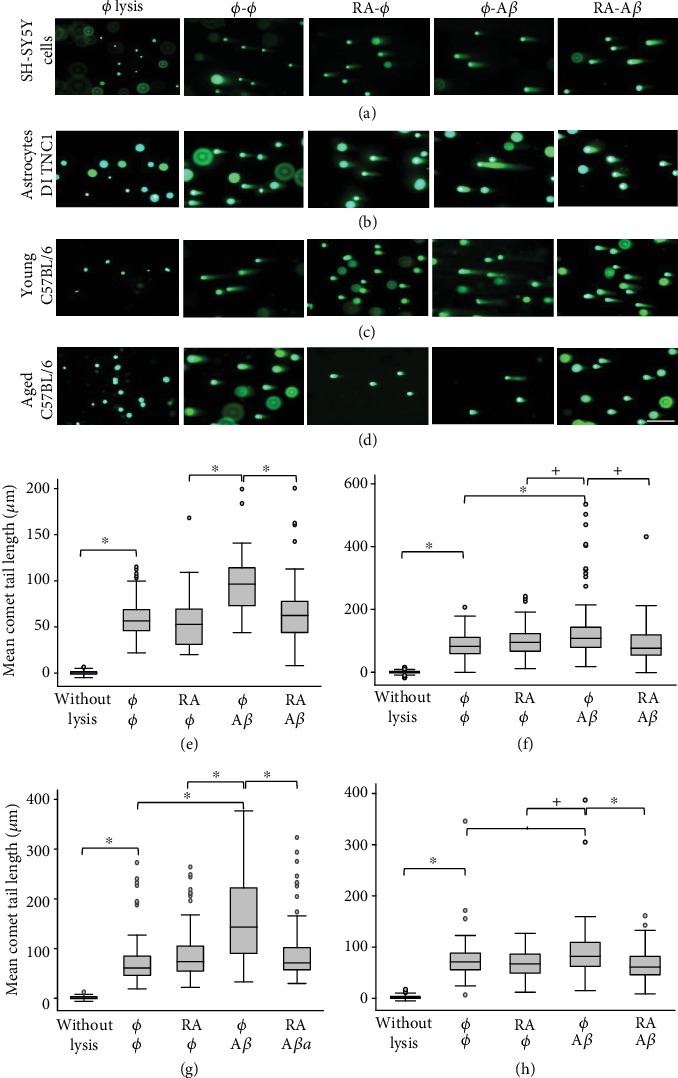
All-*trans*-retinoic acid (RA) protects against A*β*-induced DSBs in SH-SY5Y and astrocytic DI TNC_1_ cells as well as in the neocortex of young and aged male C57BL/6J mice. Representative pictures of comets with various tail lengths of (a) SH-SY5Y cells, of (b) DI TNC_1_ cells, and of cortical tissues originating from (c) young (4 months; *n* = 3 mice) or (d) aged (16 months; *n* = 3) mice following RA (5 *μ*M) and/or A*β* (20 *μ*M) *in vitro* treatments for 2 × 30 min (SH-SY5Y cells; cortical tissue) and 2 × 1 h (DI TNC_1_ cells). ø = without treatment; scale bar: 200 *μ*M. Box plots of mean comet tail lengths of (e) SH-SY5Y cells (number of cells measured: 33 < *n* < 52), of (f) DI TNC_1_ cells (55 < *n* < 72), of (g) 3 young (30 < *n* < 55), and of (h) 3 aged mice (31 < *n* < 57). ANOVA with Bonferroni correction: one experiment for SH-SY5Y cells (replicated in Figure 4(e)) and 3 for DI TNC_1_ cells; ^∗^*p* < 0.05 for all experiments or all three mice; ^+^*p* < 0.05 for 2 out of 3 mice; ^◊^*p* < 0.05 for 1 out of 3 mice.

To demonstrate that RA can protect against A*β*-induced DSBs, SH-SY5Y cells and astrocytic DI TNC_1_ cells were treated with RA half an hour before the addition of A*β* or not, for 30 min. The presence of RA before A*β* resulted in comets with mean tail lengths similar to untreated lysed or RA-treated SH-SY5Y or DI TNC_1_ cells and significantly shorter than A*β*-treated cells (Figures 1(a), 1(b), 1(e), and 1(f)). We observed a slightly more important decrease of mean comet tail length in astrocytic DI TNC_1_ cells (48%) than in SH-SY5Y cells (32%) for the RA neuroprotective treatment against A*β* (average of 3 experiments).

RA added half an hour before the A*β* treatment can protect also against DSBs in cortical tissues originating from young (4 months old; *n* = 3) and aged (16 months old; *n* = 3) C57BL/6J male mice. The resulting tail lengths were comparable in length to the untreated lysed cortical cells and to the cortical cells treated with RA alone (Figures 1(c) and 1(d)). The mean comet tail length was significantly higher in A*β*-treated cortical cells compared to all other conditions in the young as well as in the aged mice (Figures 1(g) and 1(h)). The difference in mean comet tail lengths between the A*β* treatment and other conditions (no treatment or RA treatment alone) was less important in the aged compared to the young mice, possibly due to the decreased metabolism. However, the difference between the treatments ø-A*β* and RA-A*β* was statistically significant in all 3 young as well as in all 3 aged mice. We observed a more important decrease of mean comet tail length in young mice (45%) than in aged mice (25%) for the RA neuroprotective treatment against A*β* (average of 3 experiments). There was also a global effect of age (*p* = 0.005) for all conditions grouped together in favor of a decrease of DSBs with age. Note that RA protects specifically against A*β*-induced DSBs. Indeed, SH-SY5Y cells grown in 10% FCS and treated with 100 nM etoposide for 24 h showed a significant increase of DSBs (*p* < 0.001, *n* = 35) that was not reduced significantly by the presence of 1 *μ*M RA, contrary to the significant increase of DSBs with 4 *μ*M A*β* for 24 h that was significantly decreased by the addition of RA (*p* < 0.001, *n* = 35; results not shown).

### 3.2. Neuroprotective Effect of Retinoic Acid against A*β*-Induced DSBs in SH-SY5Y Cells in comparison to Other Antioxidants

**Figure 2:**
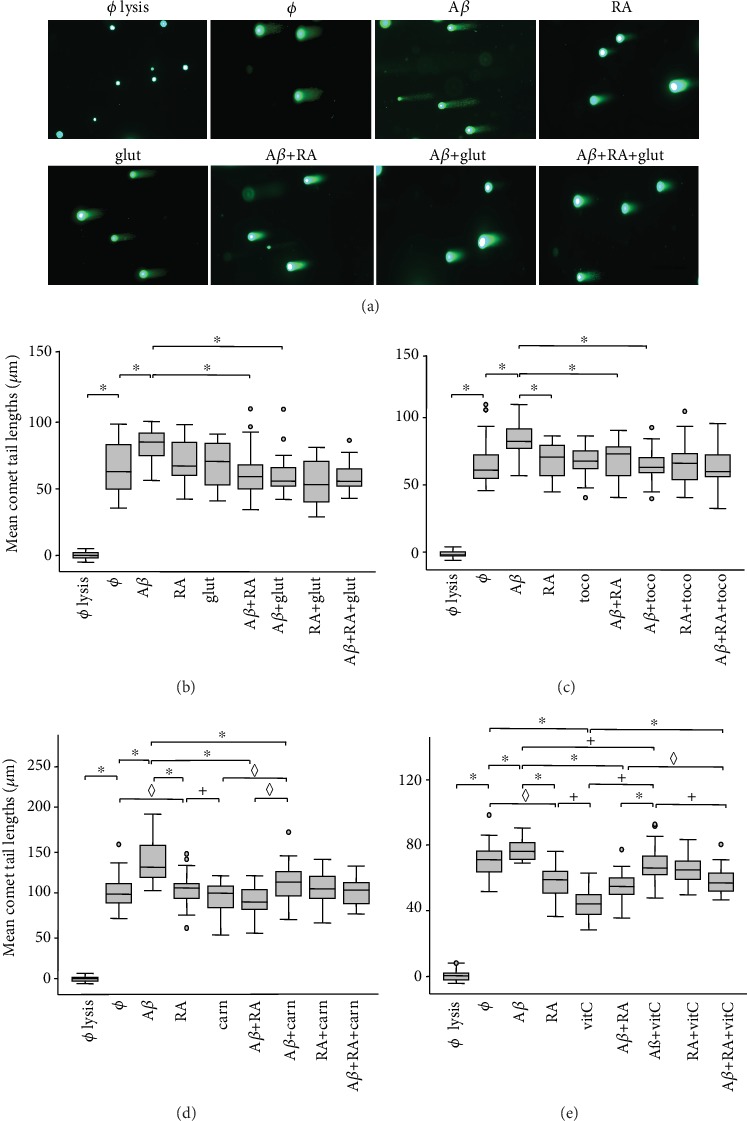
Effects of antioxidants against A*β*-induced (20 *μ*M) DSBs in SH-SY5Y cells compared to all-*trans*-retinoic acid (RA; 5 *μ*M) treatment. In (a), example of comet assay with 1 h treatments with A*β*, RA, and/or glutathione (glut). Scale bar: 200 *μ*M. In (b) to (e), data analyses: (b) glutathione (1 *μ*M), (c) *α*-tocopherol (toco; 1 *μ*M), (d) L-carnosine (carn; 100 *μ*M), and (e) vitamin C (vitC; 100 *μ*M). Box plots of mean comet tail lengths (number of cells measured: 18 < *n* < 53). ANOVA with Bonferroni correction: the experiments with L-carnosine and vitamin C were repeated 3 times (^***◊***^*p* < 0.05 for 1 experiment out of 3; ^+^*p* < 0.05 for 2 experiments out of 3; ^∗^*p* < 0.05 for 3 experiments out of 3) whereas the experiments with glutathione and *α*-tocopherol were carried out 1 time (^∗^*p* < 0.05). ø = without treatment.

SH-SY5Y cells were treated with glutathione, *α*-tocopherol, L-carnosine, or vitamin C and/or A*β* for 1 h in comparison to the RA treatments (Figure 2). If there was a significant decrease (*p* < 0.05; Bonferroni test) in average tail lengths comparing the A*β* treatment with the A*β*+RA or A*β*+glutathione treatments, there was no significant difference between RA or glutathione and no treatment. Furthermore, no additive or synergistic effects were observed by comparing the A*β*+RA or the A*β*+glutathione treatments with the A*β*+RA+glutathione treatment (Figures 2(a) and 2(b)). Similar observations were made with *α*-tocopherol (Figure 2(c)) and L-carnosine (Figure 2(d)), i.e., there were no differences between the treatments with these antioxidants and no treatment and no additive or synergistic effects. Vitamin C was able to reduce DSBs more than RA in the absence of A*β* in two-third of the experiments. However, if vitamin C significantly (*p* < 0.05) reduced DSBs compared to the controls, the A*β*-induced DSBs in the presence of vitamin C were always significantly more numerous than in the presence of RA, indicating that vitamin C was less efficient to reduce A*β*-induced DSBs than RA. Furthermore, combined treatment of vitamin C and RA showed no diminution of DSBs compared to the untreated cells (Figure 2(e)).

### 3.3. Retinoic Acid-Dependent Protein Expression in Cortical Regions of Young and Aged C57BL/6J Mice in relation to the Amyloid Cascade

**Figure 3:**
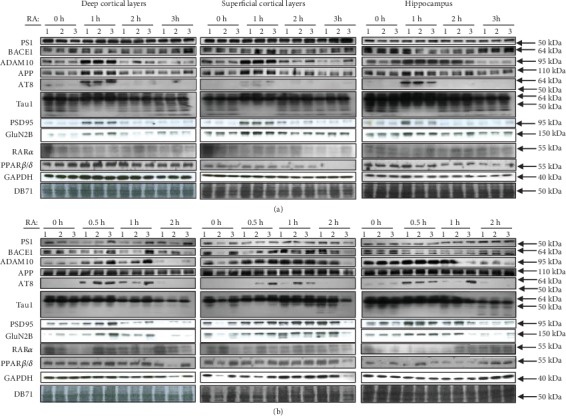
Changes in protein expression following 1 *μ*M all-*trans*-retinoic acid (RA) treatment of the deep and superficial neocortical layers, as well as of the hippocampus, of (a) three 1-month-old and (b) three 17-month-old male C57BL/6J mice. The proteins are involved in the amyloid cascade (Presenilin 1 or PS1/*γ*-secretase, BACE1/*β*-secretase, ADAM10/*α*-secretase, and APP C-terminus), in Tau phosphorylation (phospho-Tau (AT8) or unphosphorylated Tau (Tau1)), in synaptic functions (PSD95, GluN2B/NR2B), or in RA-dependent pathways (RAR*α*, PPAR*β*/*δ*). GAPDH and DB71 stainings were used to demonstrate equal loading of the Western blot gels. Overall, we observed significant increases (see Section 3.3) of ADAM10, APP, and phosphorylated and unphosphorylated Tau proteins, suggesting the activation of neuroprotective mechanisms following the RA treatment, whereas the expression of the enzymes of the amyloidogenic pathway, PS1 and BACE1, or, in most cases, of the RA receptors was not increased. Protein sizes are indicated on the right. A size range is given for Tau isoforms (AT8 and Tau1).

The effect of 1 *μ*M RA on the expression of various gene products was tested in three 1-month-old male versus three 17-month-old male C57BL/6J mice in the dissected deep and superficial neocortical layers and the hippocampus (Figure 3). The gene products belonged either to the amyloid cascade (Presenilin-1/*γ*-secretase or PS1, BACE1/*β*-secretase, ADAM10/*α*-secretase (immature form), and APP (C-terminus)), to Tau (AT8, Tau1), to synaptic markers (PSD95, GluN2B), or to receptors of the RA pathways, RAR*α* and PPAR*β*/*δ*. Their expressions were always measured in comparison to the controls, GAPDH or/and DB71 protein staining. Statistical analyses were carried out only for the neocortex and not the hippocampus, this structure being less homogenous than the neocortex. However, the results appeared to be similar. Whereas PS1 and BACE1 were not increased after 1 h RA treatment, ADAM10 and APP were increased by factors 5.5 and 1.3 in the deep cortical layers and 1.2 and 1.05 in the superficial cortical layers in young and aged mice grouped together. Significance (Wilcoxon signed-ranked test; *p* = 0.0277) was however only reached in the deep cortical layers with ADAM10 and APP. ADAM10 and APP increased in both cortical layers with age (*p* = 0.0495; two-sample Wilcoxon rank-sum (Mann-Whitney)), whereas PS1 and BACE1 diminished in both layers with age (*p* = 0.0495). These data suggest that RA decreases the amyloid cascade and the A*β* production in young and in aged mice.

Concerning the phosphorylated Tau isoforms, the main band observed with AT8 (around 60 kDa) increased after 1 h RA treatment by factors 10 and 2.2 in the young mice and 8.7 and 3.6 in the old mice for the deep and superficial cortical layers, respectively. Thus, statistical analyses showed an increase due to the RA treatment in the deep layers (*p* = 0.0214) when normalized with GAPDH (not DB71) but not in the superficial layers. The main band of unphosphorylated Tau, around 60 kDa, detected with the Tau1 antibody, increased after 1 h RA treatment by factors 1.1 and 1.1 in the young mice and 1.5 and 1.3 in the old mice for the deep and superficial cortical layers, respectively. Statistical analyses did not show a significant increase following the RA treatment, whereas a band of 70 kDa (*p* = 0.04/DB71; *p* = 0.0014/GAPDH) and one of 50 kDa (*p* = 0.0309/DB71; *p* = 0.0077/GAPDH) reached significance following the RA treatment in the deep layers only. The simultaneous increase of phosphorylated and unphosphorylated Tau indicates an overall enhanced Tau metabolism in the axons due to the RA treatment.

This observation appears to be corroborated by an increase of postsynaptic proteins PSD95 and GluN2B after 1 h RA treatment in young mice and less so in old mice in all tissues, despite the fact that a statistically significant difference in relation either to the treatment or to age was not reached.

Finally, the RAR*α* and PPAR*β*/*δ* receptors showed no changes in expression following the RA treatment independently of age, if we except a significant increase of the PPAR*β*/*δ* receptor in the deep layers after 1 h treatment with RA (*p* = 0.043). There was a significant increase of PPAR*β*/*δ* and RAR*α* receptors in old mice compared to young ones in the superficial and the deep cortical layers for no or 1 h treatment with RA and most of the time for 2 h treatment. Finally, both PPAR*β*/*δ* and RAR*α* receptors were statistically more expressed in the superficial layers than the deep layers of the neocortex.

Overall, the significant increase of proteins ADAM10, APP, and phosphorylated and unphosphorylated Tau proteins, not of PS1 and BACE1, especially in the deep cortical layers, suggests an inbalance in favor of the antiamyloidogenic pathway following the RA treatment. This appears not to be due to an increase in expression of the RAR*α* and PPAR*β*/*δ* proteins subsequently to the RA treatment, except in the deep layers for PPAR*β*/*δ*. However, a higher expression of these receptors with age and in the superficial cortical layers might play a neuroprotective role.

### 3.4. A*β*-Induced DSBs Are Transcription and Translation Dependent, Contrary to RA Protection against DSBs

**Figure 4:**
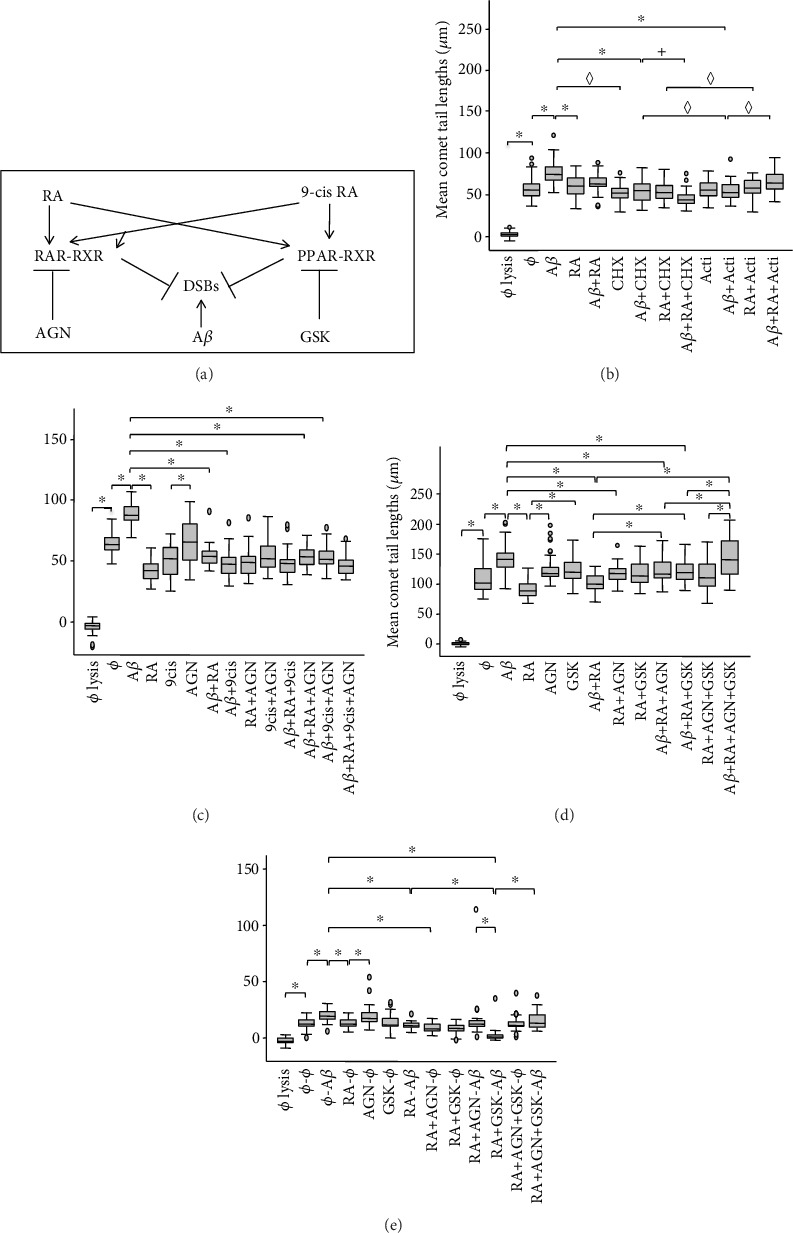
(a) Schematic representation of the RA and 9-cis RA receptors, RAR, PPAR, and RXR, and their antagonists, AGN 193109 (AGN) for RAR*α*,*β*,*γ* and GSK 3787 (GSK) for PPAR*β*/*δ*, as well as their neuroprotective effect on A*β*-induced double-strand breaks (DSBs). (b) Effects of 5 *μ*M all-*trans*-retinoic acid (RA) against A*β*-induced DSBs and of 20 *μ*M A*β* on DSB production in the presence of the transcription inhibitor actinomycin D (Acti; 1 *μ*g/mL) and of the translation inhibitor cycloheximide (CHX; 10 *μ*g/mL) in SH-SY5Y cells after a 1 h treatment. The inhibitors do not interfere significantly with the effect of RA whereas they do with A*β*. Box plots of mean comet tail lengths of SH-SY5Y cells (number of cells measured: 27 < *n* < 50). ANOVA with Bonferroni correction: the experiment was repeated 3 times (^***◊***^*p* < 0.05 for 1 experiment out of 3; ^+^*p* < 0.05 for 2 experiments out of 3; ^∗^*p* < 0.05 for 3 experiments out of 3). (c) Box plots of mean comet tail lengths of SH-SY5Y cells (31 < *n* < 53) following a 1 h treatment with RA, 9-cis RA (5 *μ*M), and/or A*β* in the presence of AGN (50 *μ*M) or not. (d) Box plots of mean comet tail lengths of SH-SY5Y cells (36 < *n* < 50) following a 30 min treatment with A*β*, RA, AGN, and GSK (10^−5^ M) and a combination of these factors. (e) Box plots of mean comet tail lengths of SH-SY5Y cells (31 < *n* < 34) following a 30 min treatment with RA, AGN, and/or GSK, and after a washing step, a second 30 min treatment with A*β* or not. (c–e) ANOVA with Bonferroni correction: ^∗^*p* < 0.05. ø = without treatment.

The next step was to investigate whether both RA-dependent pathways (Figure 4(a)) needed transcription or translation (Figure 4(b)) or which one was preventing A*β*-induced DSBs (Figures 4(c)–4(e)). To verify if the effect of RA and A*β* on DSB protection and production, respectively, involves transcription or translation, a comet assay was carried out in triplicate with SH-SY5Y cells. They were treated 1 h with RA (5 *μ*M), A*β* (20 *μ*M), and RA+A*β* in the presence of transcription inhibitor, actinomycin (1 *μ*g/mL), or of translation inhibitor, cycloheximide (10 *μ*g/mL; Figure 4(b)). No difference in DSB levels was observed between the untreated and the actinomycin- or cycloheximide-treated cells, showing that the physiological background of DSBs is transcription- and translation-independent. There was a statistical decrease in A*β*-induced DSBs comparing A*β* and A*β*+actinomycin, or A*β* and A*β*+cycloheximide, indicating that A*β* needs transcription and translation for DSB production. On the contrary, no statistically differences in DSBs were detected between RA and RA+cycloheximide or between RA and RA+actinomycin. This indicates that, at least for a 1 h treatment, DSB levels due to RA are transcription- and translation-independent and that RA can protect against A*β*-induced DSBs at the posttranslational level.

The time needed for inhibiting transcription and/or translation seems however important. Indeed, in an experiment with a 30 min treatment only (results not shown), we observed a significant increase of DSBs with A*β*, whereas, for all the other conditions (e.g., A*β*+cycloheximide or A*β*+actinomycin), the average DSB values were lower, but not significant. The differences between RA and RA+cycloheximide or between RA and RA+actinomycin were not significant, corroborating the observation that DSB levels due to short-term RA treatments are transcription- and translation-independent.

### 3.5. RA Neuroprotection against A*β*-Induced DSBs Involves Both the RAR*α*/*β*/*γ* and PPAR*β*/*δ* Pathways

To determine which RA-dependent pathways were involved in protection against A*β*-induced DSBs, we first tried to inhibit the RA-mediated neuroprotective effect against A*β*-induced DSBs with the RAR*α*/*β*/*γ* antagonist AGN 193109 (or AGN) in the presence or not of the 9-cis RA that binds not only to RARs but also to RXRs (Figure 4(a)). For this purpose, SH-SY5Y cells were treated for 30 min with 5 *μ*M RA or 9-cis RA, and/or 20 *μ*M A*β*, in the presence or not of AGN. If there was a significant decrease (*p* < 0.05; Bonferroni test) of average tail lengths comparing the RA+A*β* or 9-cis RA+A*β* treatments with the A*β* treatment, no significant difference was observed between both retinoids in the presence of A*β*. RA and 9-cis RA showed no additive or synergistic effects (Figure 4(c)). The presence of AGN was unable to prevent the neuroprotective effect of either RA or 9-cis RA, suggesting the involvement of at least another independent neuroprotective pathway. We decided to investigate the PPAR pathway (Figure 4(d)) by inhibiting PPAR, RAR, or both receptors together, in the absence or presence of RA or RA+A*β*, with AGN and/or the PPAR*β*/*δ* antagonist GSK 3787 (or GSK). We showed first that SH-SY5Y cell viability was not altered by GSK compared to the untreated control when the concentrations were between 10^−5^ and 10^−9^ M for a treatment of 30 min. However, a higher concentration (10^−3^ M) caused an important decrease of viability similar to that of digitonin (results not shown). Thus, the concentration of 10^−5^ M GSK was chosen, as it does not alter cell viability. The experiment in Figure 4(d) with 30 min treatment revealed that both pathways, the RAR and the PPAR pathways, contribute to the neuroprotective effect of RA against A*β*-induced DSBs (A*β*+RA versus A*β*+RA+AGN or A*β*+RA+GSK). Only when both pathways were inhibited together was RA unable to protect against A*β*-induced DSBs (RA+AGN+GSK versus A*β*+RA+AGN+GSK). Furthermore, both pathways appear to be active in the absence of exogenously added RA, as a significant increase was observed between the AGN or the GSK treatment and the RA treatment.

This experience was repeated but with two steps for 30 min each(Figure 4(e)). The neuroprotection step—RA, AGN, GSK, RA+AGN, RA+GSK, RA+AGN+GSK, or no treatment—inhibited or not, was, subsequently to a washing step, followed by the addition of A*β*, or not, as control. The observations showed that, as the inhibition of PPAR by GSK in the presence of RA and A*β* significantly decreased DSBs in comparison to RA-A*β*, the RAR pathway plays a major role under these experimental conditions. As no difference was observed between the treatment with AGN in the presence of RA and A*β*, and the combined RA-A*β* treatment, this also suggests that the PPAR pathway plays a role in neuroprotection against RA. The fact that the RA+GSK-A*β* treatment was significantly different compared to the ø–A*β* treatment, but not to the RA+AGN-A*β* treatment, suggests however that the PPAR pathway is less important under these circumstances. However, when both pathways were inhibited together (RA+AGN+GSK-5A*β*), neuroprotection appeared almost fully inhibited. Overall, experiments of Figures 4(d) and 4(e) showed similar results, i.e., both, the RAR and PPAR pathways, are involved in prevention against A*β*-induced DSBs and complement each other.

### 3.6. Activation of PPAR-RXR Heterodimerization following RA–A*β* Neuroprotective Treatment

With both PPAR and RAR pathways being involved in neuroprotection against DSBs, we tried next to show their activation by immunofluorescent cytochemistry and by Duolink™ *in situ* proximity ligation assay (PLA) using in both cases the same treatments. These methods were carried out to determine either colocalisation or heterodimerization of PPAR*β*/*δ* and RXR*α*/*β*/*γ* or RAR*α* and RXR*α*/*β*/*γ*.

**Figure 5:**
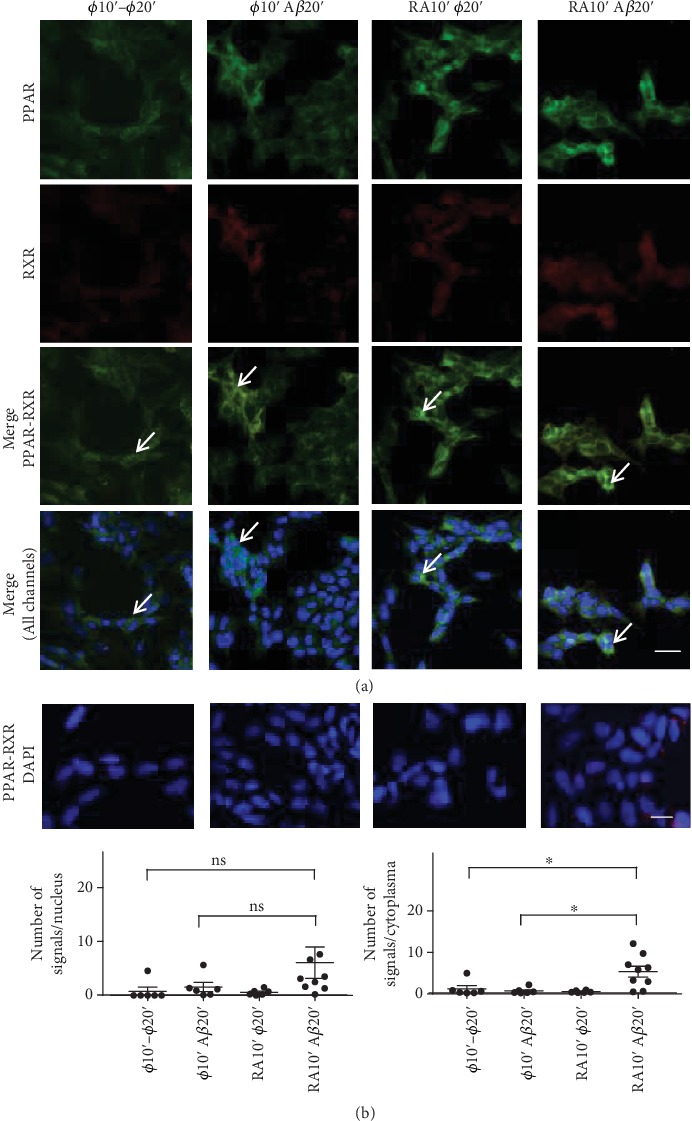
Neuroprotection experiments carried out with SH-SY5Y cells to show the activation of RA-dependent pathways by a 10 min RA treatment or not (ø), followed by a 20 min treatment with A*β* or not (ø10′–ø20′, ø10′–A*β*20′, RA10′–ø20′, and RA10′–A*β*20′). (a) By immunofluorescent cytochemistry, it was shown that PPAR*β*/*δ* (green) increased in the cytoplasm with the RA–A*β* treatment, whereas a cytoplasmic increase of the RXR*α*/*β*/*γ* (red) could not be observed. However, sites of colocalisation (white arrows, green-yellow signals) could be detected (Merge, PPAR-RXR, or all channels). Signals were too faint in the cell nucleus (DAPI). (b) Duolink™ proximity ligation assay was used to determine PPAR*β*/*δ* and RXR*α*/*β*/*γ* heterodimerization under the same conditions as in (a). A significant increase of PPAR-RXR signals was observed with the RA–A*β* treatment only in the cytoplasm when compared to the ø–ø (*p* = 0.0125) or ø–A*β* (*p* = 0.0145) treatments. Only a nonsignificant (ns) increase was observed in the cell nuclei. The number of pictures analysed is 6 to 9. Similar experiments for RAR*α* and RXR*α*/*β*/*γ* resulted in no significant differences. Scale bar for (a): 25 *μ*M and for (b): 13 *μ*M.

By immunocytochemistry (Figure 5(a)), we could observe in SH-SY5Y cells low RXR*α*/*β*/*γ* signals and increased PPAR*β*/*δ* signals in the cytoplasm, not in the cell nucleus, especially when they were treated with RA and A*β*. Moreover, colocalisation of PPAR and RXR signals could be found in the cytoplasm, notably when the cells were treated with RA and A*β*, suggesting possible PPAR-RXR interactions in agreement with PLA results. In the PLA experiments, when the SH-SY5Y cells were treated simultaneously with A*β* and RA for 30 min, no significant increases of PPAR-RXR or RAR-RXR heterodimers occurred either in the cell nucleus or the cytoplasm. However, a nonsignificant (*p* = 0.07) increased number of PPAR-RXR signals was observed with the RA and A*β* treatment compared to the nontreated cells in the cytoplasm but not in the nucleus. When we repeated the experiment with a 10 min neuroprotective step (RA or ø) followed by a 20 min A*β* treatment or not (ø–ø, ø–A*β*, RA–ø, and RA–A*β*), we detected a significant increase of PPAR-RXR signals (Figure 5(b)), not of RAR-RXR signals (results not shown), with the RA–A*β* treatment only compared to the ø–ø (*p* = 0.0125) or ø–A*β* (*p* = 0.0145) treatments in the cytoplasm. An increase was observed in the cell nucleus; however, it was not significant. The immunocytochemical and the PLA results both support enhanced interactions of PPAR-RXR within the chosen time range. This was not the case for the RAR-RXR data (results not shown). Overall, this suggests that in the presence of RA and A*β*, not of RA or A*β* alone, PPAR-RXR interactions are favored thus decreasing DSBs.

## 4. Discussion

### 4.1. Retinoic Acid Protects against A*β*-Induced DSBs

In 17 experiments, we showed that 20 *μ*M A*β*42 produced in average 101 *μ*m long comet tails, whereas no treatment, 5 *μ*M RA, A*β*42 added together with RA, and control without lysis generated mean tail lengths of 74.8, 70.2, 73.2, and 0.4 *μ*m, respectively. This clearly illustrates the effect of A*β* on DSB production and of the counteracting effect of RA, i.e., an effect that can either be reparative (RA added after A*β*) or protective (RA added at the same time or before A*β*). Whereas in a previous publication we demonstrated that RA can repair A*β*-induced DSBs [27], in the present work, we showed that RA also prevents A*β*-induced DSBs. RA can do it not only in SH-SY5Y and astrocytic DI TNC_1_ cells but also in the neocortex of young and aged C57BL/6J mice.

### 4.2. Neuroprotection against A*β*-Induced DSBs Is Mediated by Multiple Metabolic Pathways beside the RA-Dependent Pathways

All compounds studied beside RA, i.e., glutathione, *α*-tocopherol, L-carnitine, and vitamin C, were also able to decrease the level of A*β*-induced DSBs. Their neuroprotective effect was as efficient as RA in most cases, with the exception of vitamin C that was consistently less efficient. Moreover, additive or synergetic effects between RA and glutathione, *α*-tocopherol, or L-carnitine to reduce DSBs were not observed. There was even an increase of DSBs in the case of the combined treatment of vitamin C and RA. Thus, our observations suggest that overall neuroprotection against A*β*-induced DSBs is implemented by several pathways acting independently. The diminution of these compounds with age might increase the general tissular vulnerability and DSB occurrence. Antioxidants, such as vitamin C and *α*-tocopherol, are indeed known to diminish in the blood of AD patients and patients with Mild Cognitive Impairment [40]. However, to ascertain this conclusion, these experiments should be repeated in combination with other vitamins and RA and with young and old mice.

### 4.3. Retinoic Acid Decreases the Amyloid Cascade in Young and Aged Cortical Tissues

RA was shown to increase in a short time the expression of ADAM10/*α*-secretase, an enzyme known to counteract the amyloid cascade by cleaving the A*β* sequence of the APP protein [32, 41]. However, we suggest here that ADAM10 is implicated in RA neuroprotection against A*β*-induced DSBs, especially as the Presenilin-1 protein, corresponding in our experiments to the immature uncleaved form [42], and the BACE1 protein did not show an increase in expression. Furthermore, it was already shown that RA inhibits the *γ*-secretase, thus increasing APP [34]. Note that the increase of the 110 kDa APP signal suggests that, despite increased cleavage at the *α*-site, there is an enhanced APP expression due to the activated RA-dependent metabolism. Increased expression of APP in the hippocampus and various cell lines was already observed [43]. Overall, the RA treatment appears to favor the antiamyloidogenic pathway and thus a decreased production of A*β*.

The fact that the increase of ADAM10 and APP expressions reached statistical significance only in the deep cortical layers of young mice might be related to the preferential vulnerability of the superficial neocortical layers to the A*β* treatment [44, 45] and to the preferential presence in these layers of amyloid plaques [46, 47]. Significantly increased unphosphorylated as well as phosphorylated Tau signals in the deep cortical layers only, subsequent to the RA treatment, suggests an activation of the metabolism in these layers mainly, contributing possibly to their increased resistance to A*β*.

The increased expression of PSD-95 due to RA is already known [48, 49]. In this study, we showed increased RA-dependent expressions of PSD-95 and GluN2B. However, they did not reach levels of significance due to the small numbers of samples (*n* = 3).

Particularly interesting was the fact that the RAR*α* and PPAR*β*/*δ* receptors were not increased in expression following the RA treatment, if we except the PPAR*β*/*δ* receptor in the deep cortical layers. Thus, the effects of the corresponding pathways on the proteins of the amyloid cascade and on the Tau protein might occur, if not by transcription, through nongenomic effects. However, it is possible that other RA-dependent receptors, such as RAR*β* [50], are involved at the transcriptional level. Note that the higher expression of the RAR*α* and PPAR*β*/*δ* receptors with age and in the superficial cortical layers might be related to a neuroprotective role.

### 4.4. A*β*-Induced DSBs Need Transcription and Translation Whereas RA-Dependent Neuroprotection against A*β*-Induced DSBs Occurs through Posttranslational Mechanisms

We showed that RA was decreasing DSBs when transcription was inhibited for 30 min, 2 × 30 min, or 1 h. As RA was shown not to enhance RAR*α* or PPAR*β*/*δ* expression in most of the murine brain tissues studied, we can propose that RA acts at the posttranslational level by favoring receptor dimerization with RXRs, chromosome decondensation, and DSB repair or prevention. Transcription of various gene products such as ADAM10 of the antiamyloidogenic cascade (Figure 3), BRCA1 [6], or antioxidant gene products, all involved in some way in DSB prevention or repair, would also be initiated but with delayed effects. The PPAR*β*/*δ* activation might also increase the expression of neprilysin or insulin-degrading enzyme [51], thus decreasing the A*β* content and DSBs. The fast, non-transcription-dependent DSB prevention and repair mechanisms, by targeting directly sites of chromosomal unwinding, are likely to play a major role in the experiments with short-time treatments as is the case in the present paper. Contrary to RA, A*β* was not able to change DSB levels when transcription or translation was inhibited for 1 h. Indeed, it is known that the A*β*_42_ peptide specifically interacts with gene regulatory elements and causes changes in transcription [2].

### 4.5. Neuroprotection against A*β*-Induced DSBs Is Mediated by Both the RAR*α*/*β*/*γ* and PPAR*β*/*δ* Pathways

We demonstrated that RA in the presence of RAR*α*/*β*/*γ* and PPAR*β*/*δ* antagonists can protect against A*β*-induced DSBs, not only via RAR receptors but also via the PPAR*β*/*δ* receptor. 9-cis RA was used as comparison. The inhibition of both RAR*α*/*β*/*γ* and PPAR*β*/*δ* receptors with either AGN or GSK in the presence of A*β* and RA resulted in the same level of A*β*-induced DSBs as the A*β* control treatment, suggesting that in the SH-SY5Y cells, these pathways are the major ones, if not the only ones.

The PLA experiments showed a significant increase of PPAR*β*/*δ*-RXR*α*/*β*/*γ* heterodimers, not of RAR*α*-RXR*α*/*β*/*γ* heterodimers, following a double-step RA-A*β* treatment. Significance was reached in the cell cytoplasm, and only a tendency was observed for the cell nucleus. The increase of PPAR was corroborated by immunocytochemistry. This suggests that RA increases PPAR in the cytoplasm and possibly in the cell nucleus, favoring interactions with RXR and the protection against A*β*-induced DSBs. In the absence of A*β*, the increased heterodimerization was not observed. Thus, it appears that, under the specific conditions used, whereas the RAR pathway is not, no more, or not yet active, the PPAR pathway is activated or still actively promoting attempts of cell division and antiapoptotic conditions [35].

## 5. Conclusions

Overall, our data suggest that the A*β*-induced DSBs can be prevented by RAR*α*/*β*/*γ* or PPAR*β*/*δ* receptors after their shuttling in the cell nucleus in the presence of RA. In the longer term, this could also result in tilting the balance of gene expression in favor of the antiamyloidogenic pathway, via ADAM10 increased expression, thus reducing A*β* generation. We proposed previously [27] that RA is involved in the repair of A*β*-induced DSBs. We have now shown that this phenomenon appears to be complemented by RA-dependent neuroprotection. Furthermore, it is possible that, depending on the distribution and levels of RA (and other vitamins) and on the A*β* production in the aging brain, a mosaic of A*β*-induced DSBs would result and only a subset of functional important genes, such as RA-dependent genes, would be selectively protected and not other age dispensable genes. Such targeted prevention of DSBs by Adaptative Gene Expression (DSB-AGE hypothesis) would allow maintenance in an energy economical manner of genes of at least the most important survival brain functions, such as memory. In all cases, this study corroborates the observations suggesting that the RA pathways can be targets of preventive strategies.

## Data Availability

All data used to support the findings of this study are available from the corresponding author upon request.
